# Effect of Ground Waste Glass Addition on the Strength and Durability of Low Strength Concrete Mixes

**DOI:** 10.3390/ma14010190

**Published:** 2021-01-02

**Authors:** Robert Jurczak, Filip Szmatuła, Tomasz Rudnicki, Jacek Korentz

**Affiliations:** 1 Department of Road and Bridge Engineering, West Pomeranian University of Technology in Szczecin, 70-310 Szczecin, Poland; robert.jurczak@zut.edu.pl; 2 General Directorate for National Roads and Motorways, 70-340 Szczecin, Poland; fszmatula@gddkia.gov.pl; 3 Faculty of Civil Engineering and Geodesy, Military University of Technology in Warsaw, 2 Gen. S. Kaliskiego St., 01-476 Warsaw, Poland; 4 Faculty of Civil Engineering, Architecture and Environmental Engineering, University of Zielona Góra, 65-417 Zielona Góra, Poland; j.korentz@ib.uz.zgora.pl

**Keywords:** recycling, waste glass, glass powder, concrete, compressive strength, durability

## Abstract

By recycling used glass containers, we are able to recover and reuse their valuable properties, which is a way to preserve the relevant natural resources and lessen environmental burdens. For example, recycled waste glass (in the form of powder) can be used in the production of concrete. This article analyses the effect of waste glass addition on the properties of C12/15, which is used, for example, as concrete bedding material to support road drainage gutters and kerbs. Ground waste glass was used as a filler in the mix, i.e., without decreasing the amount of cement. Brown glass collected as municipal solid waste was used in this research. The research comprised an experiment prepared on the basis of the central composite design. The independent variables included water/cement ratio and the amount of glass powder, expressed as the glass to cement ratio by weight. The adopted research program mainly included the definition of the concrete compressive strength, water absorption and freeze–thaw resistance after 25 and 100 cycles of freezing and thawing. For selected systems, the characteristics of air voids in hardened concrete were also defined. The beneficial effect of ground waste glass added as a filler to the concrete mixture on the strength and durability of concrete was confirmed by the obtained test results.

## 1. Introduction

The aspects related to the reuse of waste materials, such as waste glass, in the field of construction have become an increasingly widely explored subject of scholarly articles. Although the amount of waste glass collected and recycled in the EU increases year by year, as demonstrated by the most recent data [1], there are very wide variations between the member states themselves. With an over 95% glass recycling rate in Sweden, Slovenia, and Belgium; in other countries, such as Greece and Hungary, there is great room for improvement in this respect. In Poland, the average glass recycling rate in 2017 was 62.5%, i.e., it was 13.7% lower than the value of the whole of the European Union. This means that waste glass and used glass containers are, in a majority, reused in the production of brand-new products, i.e., utilised in the most efficient way. On the other hand, ca. 37.5% of this waste is not recycled at all, i.e., it is sent directly to landfill.

Currently, waste glass is used as a raw material for the production of glass fibres used in production of thermal and sound insulation panels, mats, and lagging. For example, a very fine-grained waste glass powder is used to produce expanded glass beads added to wall coatings and mortars. Another waste glass material, namely waste flat glass, is used for the production of glass microspheres [2].

Numerous studies have been conducted on the possibility of using finely ground waste glass as a partial replacement for cement in concrete. A smaller glass particle size led to a higher compressive strength in concrete and compared to fly ash concrete, concrete containing ground glass exhibited a higher strength at both early and late ages [3]. The compressive strength test results indicated that recycled glass mortar and concrete gave better strength compared to control samples. A 20% replacement of cement with waste glass was found to be convincing considering the cost and the environment [4]. Test results [5] indicate that use of glass powder in concrete has improved the performance of concrete in strength. The addition of glass powder in the amount of 15% results in the highest increase in compression, bending and tensile strength. Many years of observations after the walls and panels were made have shown a continuous improvement in the mechanical performance and resistance chloride-ion penetration of the glass powder concrete due to its pozzolanic reactivity. The water absorption and porosity were also decreased when using glass powder [6]. The incorporation of glass as a cement replacement or even as aggregate can decrease the alkali silica reaction (ASR) effects and its efficiency is related with the replacement ratio [7]. Concrete with cement replaced by 15% and 30% glass powder exhibited the highest strength increase and correspondingly the lowest porosity [8]. However, the high-volume glass powder concrete retained distinct resistance against water and chloride ingress, due to the reduction in pore size and connectivity. Other research [9] has shown that the use of recycled glass powder (RGP) as cement replacement is feasible for a replacement level up to 10%. However, long term curing and lower particle size distribution are mandatory for the successful use of RGP with higher replacement levels without compromising the strength. Test results [10] reflect a slow and continuous pozzolanic activity of the glass powder in mixtures with enough free water available. The improvement in the mechanical strength and durability of the cementitious materials modified with glass powders can be attributed to microstructure improvement arising from the pozzolanic property of the glass powders [11].

The results of studies on the impact of glass granulate as a substitute for natural aggregate in concrete are known. The addition of 25% and 50% of crushed waste glass sand promotes a significant improvement in strength, and the optimum glass content should be 25% for the production of sustainable eco-concrete [12]. With the addition of 5, 10, 15 to 20 wt.% glass aggregate, the increase in compressive, flexural, and split tensile strength of mortar with glass sand aggregate compared to the reference mix ranging from about 11% to 29%, 3% to 14% and 20% to 23%, respectively [13]. Replacement of natural aggregate with glass aggregate results in a significant increase in concrete strength [12,13], but not in all cases. In contrast, the test results [14] showed that the greater the addition of recycled glass aggregates produced of exploded lighting materials, the less advantageous the features of the concrete obtained with its participation are. The addition of waste glass as a partial substitute to fine aggregate has been reported to improve the mechanical properties of heat resistant cementitious mortars [15]. Additionally, based on a review of several scholarly papers [16,17,18,19,20,21,22], we can state that waste glass can be used, depending on its fineness, as a partial replacement to fine aggregate or cement; in the latter case, playing a role of a binder.

Researchers expect that fly ash and slag may soon become scarce due to their increasing reuse in the production of energy, similarly to other renewable sources. It is, therefore, justified to search for alternative fine-grained materials that could be successfully used in the production of concrete. Ground waste glass can be an option of choice in these circumstances. Other than fly ash or slag, this additive is not a combustion by-product. Moreover, due to the high cost of segregating small-size waste glass items (advanced sorting systems are needed in the process), coloured waste glass seems to be a very promising option in this application. This applies in particular to lower strength classes of concrete (i.e., up to C20/25), in which various types of waste/by-products coming from various industries have been successfully used for a number of years. The primary applications of this material include bedding under road gutters and kerbs and as a road-base material. It is related to a very wide range of infrastructural investments, which are and will be implemented in Poland for many years.

In Poland, road engineers use in the design process the so-called catalogues of standard constructions [23,24], which specify the materials that should preferably be applied for the lower courses of pavement. Portland cement concrete (PCC) is used solely as a pavement surfacing material. It is not specified for other courses of the pavement structure, such as road-base course. That said, PCC could in theory be used as road-base material in pavements of bespoke design. One also cannot find a construction appropriate for bus lay-by pavements in the above-mentioned catalogues. In Poland, the minimum strength is the one and only requirement that bedding concrete for road items must satisfy according to the requirements of the relevant construction and testing codes issued by General Directorate for National Roads and Motorways (shortened to GDDKiA) [25]. The detailed design requirements applicable to PCC road-bases are given in the Polish standard no. PN-S-96014:1997 [26], which is still in force in Poland. The criteria specified in this standard for road-base concrete include compressive strength as required for C12/15 concrete and water absorption not greater than 7%. Freeze–thaw resistance of concrete is also checked after twenty-five freeze–thaw cycles according to the standard method described in the standard [26]. The test is passed if the average compressive strength of specimens subjected to cyclic freezing and thawing is not smaller than 80% of the average compressive strength of non-tested specimens.

The review of the mix specifications of concrete types currently used on the national roads in the West Pomerania province of Poland showed that C12/15 and C16/20 are the prevalent types of concrete mixes. C12/15 concrete is used to support precast gutters at the edges of road pavements and kerbs around the roundabout overrun areas and traversable roundabout islands. C16/20 concrete, in turn, is used much less frequently, predominantly as the base course of pavements in bus lay-by areas. This gives a chance to utilise in this way at least a part of the waste glass sent to landfill. With a vast majority of the research project carried out so far dealing with the use of waste glass as a partial substitute to cement and fine aggregate, the authors have decided to investigate the use ground waste glass as a filler, without decreasing the content of cement in the concrete mixture.

## 2. Materials and Methods

**Figure 1:**
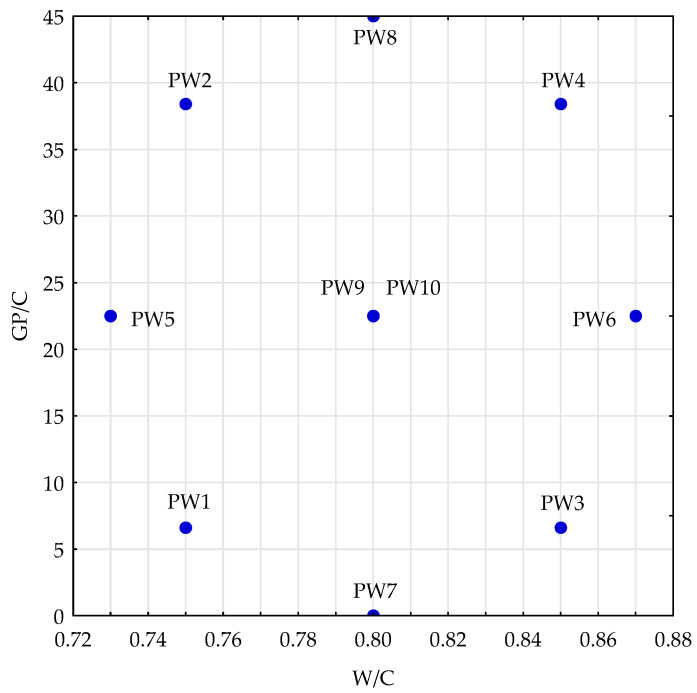
Applied central composite design.

As part of the project, an experiment plan was generated in the statistical program Statistica (the “Design of Experiments” module) [27]. A central composite design was chosen with two replications of the central point. It belongs to static, determined and poly-section plans. The layout of points in the experiment plan has been presented in Figure 1.

The tests were carried out on concrete mixes based on natural aggregate for five intermediate input values x_1_ (water to cement ratio, shortened to W/C) and x_2_ (glass powder to cement ratio, shortened to GP/C). The input values were W/C, in the range of 0.73–0.87 and GP/C, which was in the range of 0% to 45%. Nine series of specimens were produced, differentiated by the concrete mix composition. The studies were carried out for five intermediate values of input data x_1_ and x_2_ determined for standardised values equal to 0, ±1 and ±1.414. The standardised values resulting from the adopted plan were calculated into real values of variables. The coded variables and the compositions of the respective series of specimens in the adopted experiment plan are compiled in Table 1. As one can notice, the plan layouts (the concrete mix marked as PW9 and PW10) present the same combinations of input data values. Repeating the experiment for these combinations is necessary for the purpose of determining the error resulting from the output data measurement uncertainty. The composition of the reference mix without any glass added, in the experiment plan designated PW7, was adopted on the basis of the analysis of the specifications of C12/15 concrete used on national roads in the West Pomerania province of Poland.

The constants were the type and class of cement, fine and coarse aggregate types, method of adding the ingredients, mixing method and time and method of compacting fresh concrete, curing time, and curing conditions and, last but not least, the test apparatus.

**Figure 2:**
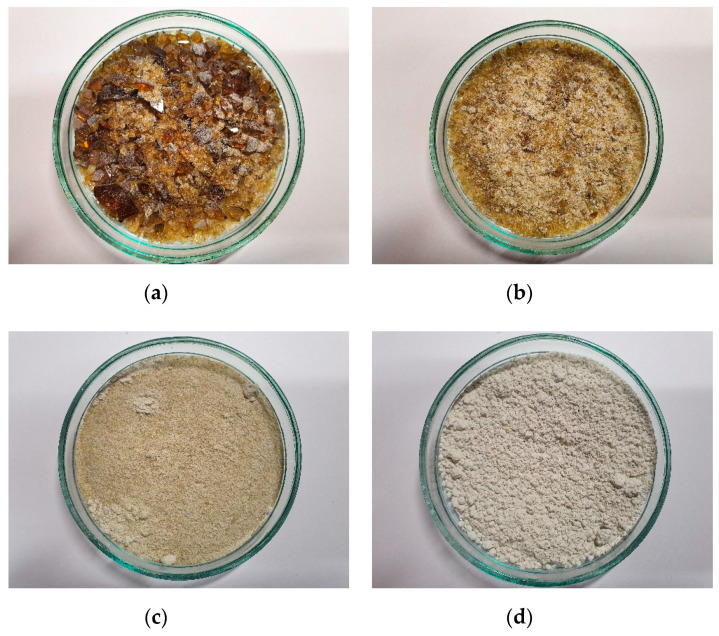
Crushing stages: (**a**) initially crushed; (**b**) crushed in crusher; (**c**) crushed in crusher and ground in mill; (**d**) crushed in crusher, ground in mill, and powdered in micro-Deval apparatus.

Brown glass was chosen for this experimental research. It was cleaned and dried and then crushed and powdered (Figure 2). The initial crushing took place in a laboratory crusher and mill (Figure 2a–c). For final crushing, the micro-Deval apparatus was used (Figure 2d). The density of ground glass used for the test was 2.515 g/cm^3^ and the density of cement was 3.102 g/cm^3^. The Blain air permeability test was used to measure the specific surface area and ground glass was found to have a greater specific surface than cement (4737 cm^2^/g compared to 3728 cm^2^/g).

The compositions of prepared mixes are given in Table 2. A change in the water/cement ratio and a different amount of the addition of glass powder result in the fact that the recipes presented in the table allow for obtaining concrete mixes of various volumes.

The mix included 200 kg of Portland cement CEM I 42.5R manufactured by Cemex Gdynia, in which cement clinker was the only cementitious material (this eliminated the need to introduce additional variables). Fine-grained aggregate was 0/2 mm sand from Bielinek sand and gravel pit. The percentage of sand contained in the aggregate mix was 50.6%. Additionally, 2/8 mm and 8/16 mm gravels from Ognica pit were used as coarse aggregate.

## 3. Results and Discussion

### 3.1. Fresh Properties

The prepared concrete mixes were tested for the basic properties including:

- Consistency, determined with the slump cone test according to EN 12350-2 [28];
- Air content determined with the pressure gauge method according to EN 12350-7 [29];
- Density according to EN 12350-6 [30].

The results of concrete mix tests are given in Table 3 below.

The consistency and air content were measured once, 15 minutes after water was added to the mix. Two of the tested mixes, namely PW4 and PW6, attained S2 consistency and the other ones fell in the consistency class S1. The total air content in the mixes ranged from 4.0–5.1%. The density of concrete mix oscillated between 2219 kg/m^3^ and 2273 kg/m^3^. Taking into consideration the measurement uncertainties, one can conclude that the addition of glass powder with an identical water/cement ratio does not impact the concrete mix air content and its consistency. The differences in the results of air content and concrete mix density are comparable and smaller than the measurement uncertainty.

### 3.2. Hardened Properties

#### 3.2.1. Compressive Strength

**Figure 3:**
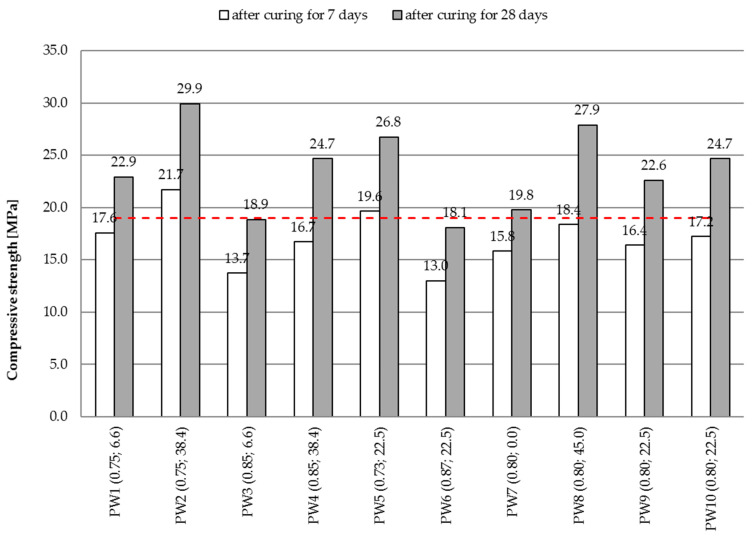
Compressive strength of concrete after curing for 7 and 28 days.

Out of each concrete mix, a series of six specimens was created for determining the compressive strength. To determine the strengths of the specimens, compression testing machines (walter + bai, Löhningen, Switzerland) were used. Compressive strength was determined after 7 and 28 days of curing the specimens in water at the temperature of 20 ± 2 °C. The tests were carried out on three 150 mm cubes, according to the procedure described in EN 12390-3 [31]. The Figure 3 represents the obtained average compressive strengths. The dashed line indicates the minimum compressive strength required to classify in C12/15 class. Additionally, values x_1_ (W/C) and x_2_ (GP/C) are given in parentheses next to the concrete mix symbol.

The compressive strength results varied strongly, in the range of 18.1–29.9 MPa. The assumed compressive strength to satisfy the requirement of the C12/15 strength class according to EN 206 after curing for 28 days [32] was obtained for seven out of nine tested concrete mixes. The highest compressive strength after curing for 28 days (in the range of 26.8–29.9 MPa) was obtained by the mixes with the lowest values of W/C ratio and different proportions of waste glass added during production (mixes designated PW2, PW5 and PW8). It was higher than the compressive strength of the reference mix without ground glass (designated PW7) by 7.0–10.1 MPa (35.4–51.0%). The lowest values of compressive strength, after curing for 28 days, were obtained by mixes with the highest W/C ratio (designated PW3 and PW6), which failed to satisfy the criteria of the adopted strength class. Furthermore, the strength parameters of five tested mixes (PW2, PW4, PW5, PW8 and PW10) satisfied the C16/20 strength class criteria. It is also worth noting that PW2 and PW5 mixes attained the assumed strength class after only 7 days of curing. Referring to the provisions of the applicable Polish code [25] relating to concrete mixes for casting strip footings, only two mixes, viz. PW3 and PW6, failed to attain the minimum compressive strength. The remaining mixes are therefore suitable as a bedding material to support road gutters and kerbs.

From the analysis of the 7 day and 28 day compressive strength values, it follows that the increase in strength of the control concrete mix by 25.1% was much smaller than that obtained for the mix containing waste glass. The highest levels of strength increase, viz. from 30.5% to 51.9%, were noted for the mixes containing waste glass. As we have observed, with the same water-to-cement ratio, the higher the proportion of waste glass, the higher the increase in the compressive strength of the mix.

#### 3.2.2. Water Absorption

**Figure 4:**
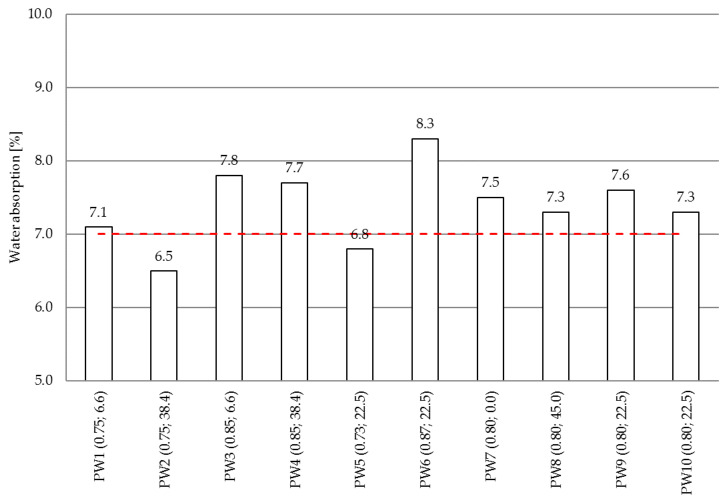
Water absorption of the tested concrete mixes.

Water absorption of the tested mixes was determined according to PN-B-06250:1988 [33]. Four 100 mm cubes were prepared for each mix specification, which after 28 days of immersion curing were dried to constant weight at a temperature of 105 °C. Then, the specimens were immersed in water, left to fully saturate and weighed once again. The average water absorption values are represented in the Figure 4. The dashed line indicates the limit according to [26].

According to the obtained water absorption test results, the absorption criterion, as per PN-S-96014:1997 [26], was satisfied by only two of the tested concrete mixes, namely PW2 and PW5. These values were in the range of 6.5–8.3% (7.4% being the average value). The lowest and highest values were obtained for PW2 and PW6 mixes, respectively. As it can be seen, mixes with the same value of the water/cement ratio, viz. 0.8 mixes no. PW7–PW10, and with different proportions of waste glass have comparable absorption capacity by weight. On the other hand, different absorption capacities were obtained for mixes designated PW1 and PW2 containing 6.6% and 38.4% of waste glass powder, respectively, despite the same (yet lower than in the case of mixes PW7–PW10) W/C ratio, which in the case under analysis was 0.75.

#### 3.2.3. Freeze–Thaw Resistance after Saturation in Water

Freeze–thaw resistance of concrete was tested according to PN-B-06250:1988 [33] with the ordinary method, procedures no. F25 and F100. Twenty-four 100 mm cubes were moulded from each of the tested mixes. The produced specimens were then immersed in water for 28 days. Next, six specimens of each mix type were weighed and subjected to twenty-five freeze–thaw cycles. The remaining six specimens were left immersed in water. After a specific number of freeze–thaw cycles (25 and 100), the specimens were weighed and assessed visually. The compressive strength of all the specimens was determined as the last step of the test procedure. The results of the freeze–thaw resistance after saturation in water are given in Table 4 and Table 5 below.

Based on the frost resistance criteria of PN-B-06250:1988 [33], the weight variation must not exceed 5% and the loss of compressive strength must not be higher than 20%, as compared to the specimens made of the same mix that were not subjected to cyclic freezing and thawing. Moreover, the specimens must not show cracking after the test.

The freeze–thaw resistance criteria were satisfied by all the mixes subjected to a standard procedure [33] comprising twenty-five freeze–thaw cycles. Distress of the surfaces or edges was not observed in any of the tested specimens. The weight loss did not exceed 0.1%, which is less than the allowable limit of ΔG = 5%. The decrease in strength after freezing and thawing cycles was in the range of 0.4–9.0% (as compared to the non-tested specimens), which is below the allowable limit of ΔR = 20%. This means that the tested mixes satisfy and exceed the F25 rating criteria by a wide margin. Based solely on the results obtained on the specimens subjected to twenty-five freeze–thaw cycles, we can conclude that all the tested concrete mixes satisfy the rating criteria of [26].

**Figure 5:**
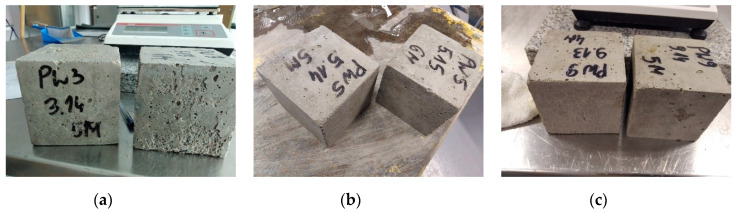
Specimens of concrete: PW3 (**a**), PW5 (**b**), and PW9 (**c**) after 100 freeze–thaw cycles.

Other than in the case of 25 freeze–thaw cycles, 100 cycles caused an average increase in the specimen weight by 0.3%. Furthermore, the higher number of cycles caused a greater decrease in the compressive strength, namely by 3.0 to 45.0%. Only two mixes showed no observable distress: PW2 and PW5 (Figure 5b). By satisfying the average decrease in strength and weight variation criteria, the specimens meet the F100 frost resistance rating criteria. Cracking was noted only in the specimens made from the PW4 mix. In addition, an average decrease in strength by a staggering 45% was obtained for this mix, which was the greatest value among all the test results. Average drops in the compressive strength in excess of 20% were obtained for the mixes designated PW3 (Figure 5a), PW6 and PW7. This being so, they failed to satisfy the F100 class rating criteria. In the case of PW1, PW8, PW9 (Figure 5c) and PW10 mixes, spalling and hairline cracking was observed, despite smaller, i.e., below 20% average drops of compressive strengths. The average drop of strength obtained for the reference mix designated PW7 exceeded the allowable limit defined by the F100 rating criteria. Considering the intended application on the tested mixes, it must be noted that a high freeze–thaw resistance was not expected of them. That said, when specifying freeze–thaw durable mixes care must be taken to specify an appropriate proportion of ground waste glass added to the mix.

#### 3.2.4. Air Void Analysis

**Figure 6:**
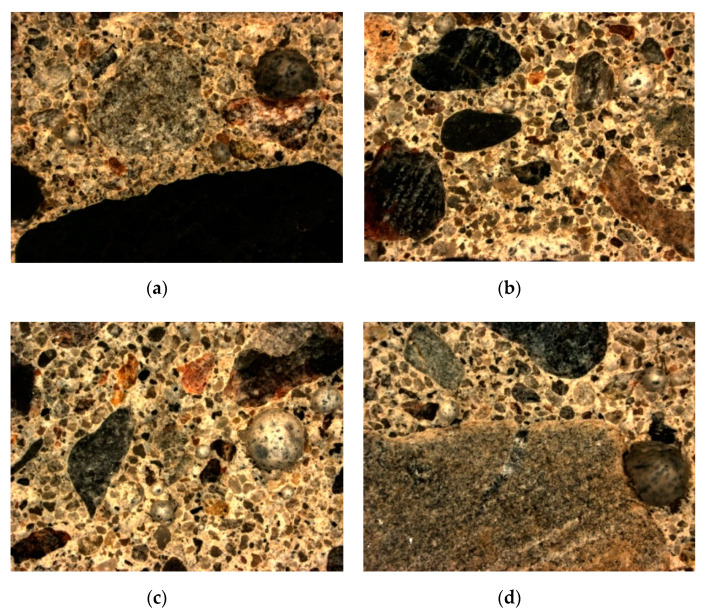
The image of the tested concrete samples after cutting: (**a**) PW5, (**b**) PW7, (**c**) PW8 and (**d**) PW9.

Defining the characteristics of air voids in hardened concrete was carried out in accordance with the EN 480-11:2008 standard [34]. Specimens for the research-dimensions 150 mm × 100 mm × 20 mm were cut out of cubes (side length: 150 mm) formed in a laboratory. Subsequently, the specimens were subjected to the process of sanding, polishing, and contrasting in order to achieve a smooth surface of the polished section. The measurement of the characteristics of the air voids was performed applying the microscope method with the use of the computer automatic image analysis system Nikon SMZ1270 Navitar. Four concretes marked as PW5, PW7, PW8 and PW9 were selected for the studies of the characteristics of air voids (Figure 6). The concretes (PW7, PW8 and PW9) were selected for the purpose of assessing the impact of the addition of glass powder (with an identical water/cement ratio equal to 0.80) on their air void characteristics. An attempt was also made to compare the spacing of the air voids in hardened concrete with an identical average amount of glass powder in concrete (22.5% in reference to the mass of the cement) but with a different water/cement ratio. As a result of the above, the concrete marked as PW5 was selected.

Concretes marked as PW5, PW8 and PW9 contain the addition of glass powder and demonstrate significant compressive strength and resistance to cyclical freezing and thawing. Concrete marked as PW7, in turn, is control concrete, which does not include the addition of glass powder. It meets the minimum requirements associated with the concrete strength class, but it does not meet the requirement of the F100 degree of freeze–thaw resistance. The most frequently adopted parameter for the assessment of the freeze–thaw resistance of concretes is the air void spacing factor *L* (the average distance to the nearest air void), which should not be larger than 0.2 mm. Another, also important parameter is the minimum air content in air voids smaller than 0.3 mm, marked as *A_300_*, which should equal at least 1.5%. The data obtained as a result of the automatic analysis of the image from four specimens of the surface from each spacing of concrete air voids selected for the analysis allowed for calculating the total content of air (*A*), the specific surface area of the air voids (α), the air void spacing factor (*L*) and the content of micro-air-voids with a diameter lower than 0.3 mm (*A_300_).* The obtained average values of the analysed parameters, defined according to the standard [35], in particular specimens are presented in Table 6. Unfortunately, for the control concrete (PW7), it was not possible to prepare specimens for the analysis of the air void structure. The specimens were falling apart during cutting and sanding. The authors did not obtain the appropriate surface of the prepared polished section, which is a necessary condition of obtaining correct results of the measurement of the air void characteristics. The reason for that may be the higher number of air voids, including ones of big sizes. The reason for the occurrence of big air voids was the hindered compacting of the concrete mix.

**Figure 7:**
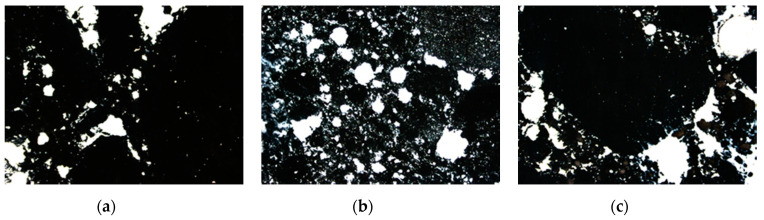
The image of the microstructure of air voids in the analysed concrete specimens: (**a**) PW5, (**b**) PW8, and (**c**) PW9 (air voids marked with white). The air void spacing meets the requirements.

While analysing the achieved results of marking the characteristics of the air void spacing in selected concretes (PW5, PW8 and PW9), it is possible to conclude that they meet the requirements in reference to concrete utilized in XF conditions. According to the standard [32], concretes in the XF exposure classes are exposed to the aggressive impact of freezing/thawing without de-icing agents (XF1) or with de-icing agents (XF2–XF4). The air void spacing factor in concrete was between 0.07 and 0.11 mm, whereas the specific surface area of the air voids was from 18.7 to 22.1 mm^−1^. Differentiated results were obtained for the total air content, unlike the results of the analysis of air content in the concrete mix, defined using the pressure method (from 5.8% to 13.2%). The total air content in the hardened concrete fits within the range of 10.9–18.2%. Such a high air content is probably the result of the insufficient compacting of the specimens. The highest value of the air void spacing factor (*L*) and the lowest content of micro-air-voids (*A_300_*) were demonstrated by the concrete marked as PW8, which included the highest amount of the addition of glass powder. In the case of an identical water/cement ratio equal to 0.80, one can notice that in concrete PW8, along with the increase in the amount of glass powder (from 22.5% to 45.0%), there was a decrease in the total content of air and micro-air-voids (*A_300_*); whereas in concrete marked as PW5, with the water/cement ratio lower than in concrete PW9 (but with identical content of the addition of glass powder), one observes lower total content of air and of air voids with a diameter lower than 0.3 mm and a lower specific surface area of the air voids (α). Figure 7 presents the characteristic images of the microstructure of air voids in the analysed concrete specimens. As a result of colouring the surface of the polished section black and of filling all the air voids with zinc paste, the contrast image required in automatic analysis was obtained.

For the purpose of designating the spacing of air voids, a calculation model was adopted, which assumes the occurrence of air voids of a certain determined diameter. It is a transitional model between the actual state and Powers’ model. The measured chords are classified into appropriate length ranges, and subsequently, the number of chords in a given length class is determined and multiplied by the volume of a single air void with the diameter equal to the top limit of a given class. The size distribution of air voids recorded during the study conducted on four specimens has been presented in Figure 6.

**Figure 8:**
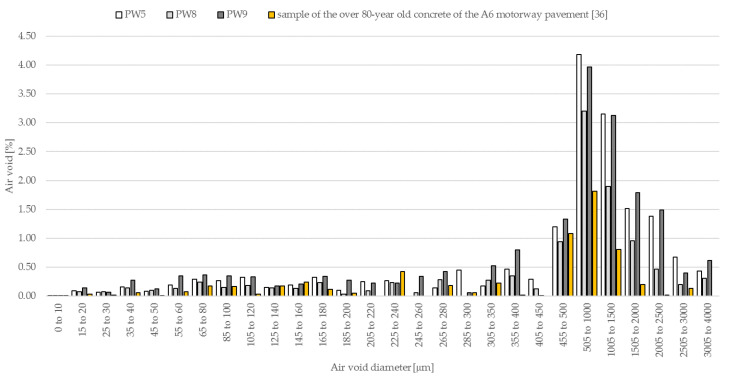
The distribution of air void diameters in the analysed concrete and sample of the over 80 year-old concrete of the A6 motorway pavement [36].

As was demonstrated in the distribution of air content as a function of the air void diameter (Figure 8) in hardened concrete (PW5, PW8 and PW9), there are both air voids of desired dimensions of 10–100 µm, as well as much bigger air voids exceeding 0.5–1.0 mm, which do not have a significant impact on the improvement of freeze–thaw resistance.

### 3.3. The Effect of Glass Powder on the Compressive Strength and Durability of Concrete

The results were subjected to a statistical analysis in the software program Statistica [27]. First, the Barlett’s test was performed to check the equality of variances. The equality of variances was confirmed at the significance level of 0.05, based on the obtained F-statistics (critical significance level *p*). The variance equality hypothesis was also found to be true through the Levene and Brown–Forsyth tests. The analysis of data also included checking the significance of the effect of input data (W/C and GP/C) on the values of the output variable (28 day compressive strength). The Fisher–Snedecor test was employed for this. The values of significance levels *p* below 0.05 indicate a significant effect of the water–cement ratio and the amount of added glass powder (in relation to the weight of cement) on the 28 day compressive strength of the tested mixes. In the further part of the statistical analysis, a model representing the effect of independent variables on the 28 day compressive strength was built on the basis of the output data of the experiment carried out according to the central composite design. A linear-quadratic model with two-way interactions was adopted in the first place. Insignificant components were then left out one by one through the analysis of the results in Statistica [27]. Finally, the factors with the greatest effect on the strength of the tested mixes were established, namely the ratio of waste glass and cement by weight followed by the water–cement ratio.

The regression function was verified on the standardised values ${\hat{x}}_{i}$ (independent variables: W/C and GP/C) for the following approximating Equation (1):

(1) $$z=A_{0}+A_{1}{\hat{x}}_{1}+A_{2}{\hat{x}}_{1}^{2}+A_{3}{\hat{x}}_{2}+A_{4}{\hat{x}}_{2}^{2}+A_{5}{\hat{x}}_{1}{\hat{x}}_{2}$$

**Figure 9:**
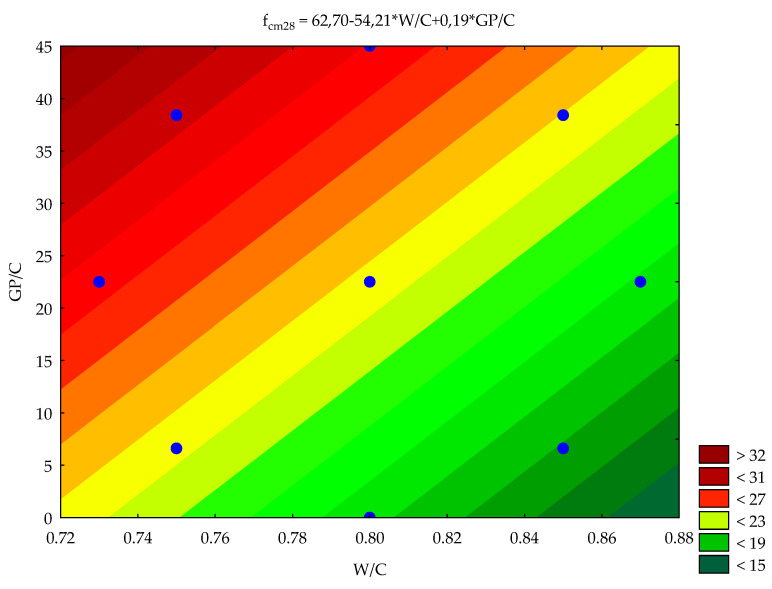
Contour diagram of the relationship between compressive strength and the amount of glass powder and water–cement ratio.

The constants *A_i_* (regression coefficient) of this equation were obtained with the Gauss–Newton method of estimation. Using the t-Student test factors, *A_2_*, *A_4_* and *A_5_* were excluded as insignificant at the significance level of 0.05. The correlation factor *R* was 0.96. In the case of two input data values (W/C and GP/C), it is possible to develop the contour diagram that presents the response surface of the object of studies. The values of the predicted 28 day compressive strength are given in Figure 9. Blue dots have been marked on the surface of the contour diagram. These are measured values introduced into the plan.

Linear relationships were obtained between the 28 day compressive strength and both of the analysed parameters, i.e., waste glass to cement ratio by weight and the water–cement ratio. From the results of the statistical analysis of the mechanical parameters obtained in the tests, it can be seen that the compressive strength increased with the increasing proportion of glass powder and the decreasing level of the water–cement ratio. The tests confirm how strongly the compressive strength depends on the water–cement ratio of the concrete mix. Within the analysed ranges, the effects of glass powder and water–cement ratio on the freeze–thaw durability of concrete are not statistically significant.

## 4. Conclusions

The tests carried out as part of this research and the analysis of the obtained result allow us to draw the following conclusions:

1. Glass powder changes the properties of low-strength concrete mixes (as compared to the control mix PW7 with the same water–cement ratio and without ground waste glass addition) as follows:
  - Improves the 28 day compressive strength by ca. 20% and 40% when added at the proportions of 22.5% and 45%, respectively;
  - Does not increase water absorption;
  - Improves the freeze–thaw durability of concrete (without the presence of de-icing agents), which is testified by compliance with the requirements given for F100 concretes designated PW8–PW10.
2. Except for PW3 and PW6, all of the tested mixes satisfied the requirements of [25] in terms of the assumed strength class and can be used for casting of footings to support kerbs and road gutters placed aside the road pavements.
3. Mixes PW2 and PW5, in addition to the above-mentioned requirements, also satisfy the compressive strength, water absorption and freeze–thaw resistance criteria defined for concrete class F25 in the Polish standard no. PN-S-96014:1997 [26], and thus can be used as road-base material under bus lay-bys and for bespoke design road pavements.
4. The average parameters of the microstructure of air voids according to the standard [35] in specimens of concrete marked as PW5, PW8 and PW9 confirm that they meet the requirements that refer to the air void spacing factor and the content of micro-air-voids with a diameter lower than 300 μm for concrete utilized in an XF aggressive environment and correspond with the results of freeze–thaw resistance.

Therefore, it is possible to produce lower strength concrete mixes containing ground waste glass in the role of filler, i.e., not as a partial substitute to cement. It remains to say, based on the study results, that the prospects for recycling waste glass as a component of concrete mixes are promising. This opinion must, however, be verified by further testing, especially using higher strength mixes. Such verification should cover, in particular, the durability of concrete, including resistance to the action of de-icing salts.

## Figures and Tables

**Table 1:** List of coded and actual variables.

Mix Symbol	Coded Variables		Actual Variables	
			W/C (x_1_)	GP/C (x_2_)
PW1	−1	−1	0.75	6.6
PW2	−1	1	0.75	38.4
PW3	1	−1	0.85	6.6
PW4	1	1	0.85	38.4
PW5	−1.414	0	0.73	22.5
PW6	1.414	0	0.87	22.5
PW7	0	−1.414	0.80	0.0
PW8	0	1.414	0.80	45.0
PW9	0	0	0.80	22.5
PW10	0	0	0.80	22.5

**Table 2:** Specifications of the tested concrete mixes.

Mix Symbol	The Composition of the Concrete Mix According to the Adopted Plan [kg]					
	CEM I 42.5 R	Sand 0/2	Gravel 2/8	Gravel 8/16	Water	Glass Powder
PW1	200	900	430	450	150	13.2
PW2					150	76.8
PW3					170	13.2
PW4					170	76.8
PW5					146	45.0
PW6					174	45.0
PW7					160	0.0
PW8					160	90.0
PW9					160	45.0
PW10					160	45.0

**Table 3:** Consistency, air content and density of the tested concrete mixes.

Mix Symbol	W/C Ratio	GP/C Ratio	Slump Cone [mm]	Air Content, [%]	Density, [kg/m^3^]
PW1	0.75	6.6	20	4.0	2244
PW2	0.75	38.4	10	4.9	2245
PW3	0.85	6.6	20	5.0	2219
PW4	0.85	38.4	50	4.1	2242
PW5	0.73	22.5	10	4.8	2260
PW6	0.87	22.5	70	4.2	2237
PW7	0.80	0.0	20	4.7	2241
PW8	0.80	45.0	30	5.1	2273
PW9	0.80	22.5	30	5.0	2237
PW10	0.80	22.5	30	4.8	2245

**Table 4:** Results of the F25 freeze–thaw test carried out on the tested concrete mixes.

Mix Symbol	Freeze–Thaw Resistance Criteria According to PN-B-06250:1988 [33]		
	Average Decrease of Strength ΔR, %	Weight Variation after Freeze–Thaw Cycles ΔG, %	Appearance, Presence of Cracks
PW1	5.9	0.04	none
PW2	2.7	0.03	none
PW3	1.0	0.05	none
PW4	9.0	0.01	none
PW5	2.0	0.04	none
PW6	4.9	0.05	none
PW7	2.3	0.08	none
PW8	6.8	0.02	none
PW9	1.5	0.06	none
PW10	0.4	0.05	none

**Table 5:** Results of the F100 freeze–thaw test carried out on the tested concrete mixes.

Mix Symbol	Freeze–Thaw Resistance Criteria According to PN-B-06250:1988 [33]		
	Average Decrease of Strength ΔR, %	Weight Variation after Freeze–Thaw Cycles ΔG, %	Appearance, Presence of Cracks
PW1	8.6	−0.21	hairline cracks
PW2	5.6	−0.04	none
PW3	22.5	−0.12	scaling and spalling
PW4	45.0	−0.92	partial and full depth cracking
PW5	3.0	−0.06	none
PW6	24.6	−0.33	cracking
PW7	24.1	-0.35	scaling and cracking
PW8	5.0	−0.15	hairline cracks
PW9	12.3	−0.18	spalling
PW10	10.5	−0.17	spalling

**Table 6:** The average values of the parameters characterizing air voids and their spacing.

Parameter	Unit	PW5	PW8	PW9
Total air content, *A*	%	16.58	10.85	18.20
Specific surface of the air void system, *α*	mm^−1^	18.73	21.93	22.13
Spacing factor, *L*	mm	0.09	0.11	0.07
Micro air-void content, *A_300_*	%	3.37	2.30	4.31

## Data Availability

Not applicable.
